# Feasibility of Recruiting Families into a Heart Disease Prevention Program Based on Dietary Patterns

**DOI:** 10.3390/nu7085323

**Published:** 2015-08-21

**Authors:** Tracy L. Schumacher, Tracy L. Burrows, Deborah I. Thompson, Neil J. Spratt, Robin Callister, Clare E. Collins

**Affiliations:** 1 Faculty of Health and Medicine, University of Newcastle, Callaghan, NSW 2308, Australia; E-Mails: Tracy.Schumacher@uon.edu.au (T.L.S.); Tracy.Burrows@newcastle.edu.au (T.L.B.); Neil.Spratt@newcastle.edu.au (N.J.S.); Robin.Callister@newcastle.edu.au (R.C.); 2 Priority Research Centre in Physical Activity and Nutrition, University of Newcastle, Callaghan, NSW 2308, Australia; 3 Hunter Medical Research Institute, New Lambton, NSW 2305, Australia; 4 USDA/ARS Children’s Nutrition Research Centre, Baylor College of Medicine, Houston, TX 77030, USA; E-Mail: dit@bcm.edu; 5 Priority Research Centre for Translational Neuroscience and Mental Health, University of Newcastle, Callaghan, NSW 2308, Australia; 6 Hunter New England Local Health District, New Lambton, NSW 2305, Australia

**Keywords:** cardiovascular diseases, diet, health education, prevention

## Abstract

Offspring of parents with a history of cardiovascular disease (CVD) inherit a similar genetic profile and share diet and lifestyle behaviors. This study aimed to evaluate the feasibility of recruiting families at risk of CVD to a dietary prevention program, determine the changes in diet achieved, and program acceptability. Families were recruited into a pilot parallel group randomized controlled trial consisting of a three month evidence-based dietary intervention, based on the Mediterranean and Portfolio diets. Feasibility was assessed by recruitment and retention rates, change in diet by food frequency questionnaire, and program acceptability by qualitative interviews and program evaluation. Twenty one families were enrolled over 16 months, with fourteen families (*n* = 42 individuals) completing the study. Post-program dietary changes in the intervention group included small daily increases in vegetable serves (0.8 ± 1.3) and reduced usage of full-fat milk (−21%), cheese (−12%) and meat products (−17%). Qualitative interviews highlighted beneficial changes in food purchasing habits. Future studies need more effective methods of recruitment to engage families in the intervention. Once engaged, families made small incremental improvements in their diets. Evaluation indicated that feedback on diet and CVD risk factors, dietetic counselling and the resources provided were appropriate for a program of this type.

## 1. Introduction

The World Health Organization reported that 17.5 million deaths were attributed to cardiovascular disease (CVD) in 2012 [1]. CVD risk factors include non-modifiable and modifiable factors, including genetic predisposition, metabolic conditions and lifestyle behaviors [2]. Offspring of parents with CVD are at increased risk due to shared genetic profiles and lifestyle behaviors [3]. In the Framingham Study, offspring with at least one parent with premature CVD had an increased age-adjusted risk of 2.3–2.6 (odds ratio) of developing CVD [4]. The Bogalusa study found that children with at least one parent with coronary artery disease (CAD) had a higher mean body mass index (1.22 kg/m^2^), total and LDL cholesterol (0.11 mmol/L, 0.14 mmol/L) and higher systolic blood pressure (1.63 mmHg) compared than children with no parental CAD [5].

Dietary patterns are an important lifestyle factor influencing the development of CVD [6,7]. Dietary patterns and eating habits are fostered within families, with younger family members modelling consumption patterns of older family members [3,8]. The Mediterranean diet is an eating pattern associated with lower risk of CVD [6], and the Portfolio diet has been shown to be efficacious in lowering CVD risk factors such as the ratio of total cholesterol to HDL cholesterol and serum triglycerides [9,10]. The Mediterranean diet is high in vegetables, fruit, nuts and olive oil with moderate intakes of fish [6]; the Portfolio diet combines foods with lipid-lowering efficacy such as soluble fibers, plant sterols and nuts [7]. Both these food-group based dietary patterns have been shown to be more effective in improving lipid profiles than diets that emphasize specific nutrient intakes such as low fat or cholesterol diets [11,12] and these food based approaches form the basis of current dietary guidelines for CVD prevention [13].

Families recruited into a dietary intervention study on the basis of one member having had an adverse CVD event, or being assessed as at high risk of CVD, may be more receptive to changing their diet. To test this hypothesis, the current study investigated: (1) the feasibility of recruiting and retaining families at increased risk of CVD into a dietary intervention program targeting alignment of existing eating patterns with heart health recommendations; (2) the dietary changes made; and (3) the acceptability of the dietary CVD prevention program.

## 2. Experimental Section

Families were eligible to participate in the “Love your Food, Love your Heart, Love your Family” (FHF) study if at least one member (index recruit aged 18–70 years) had experienced an adverse CVD event or was classified as being at moderate-to-high risk using Australian cardiovascular risk charts (aged 18–80 years) [14], had no other medical conditions affecting dietary intake, and had internet access. Written informed consent was obtained from all family members, with those < 18 years giving assent and having parental consent. Ethics approval was obtained from University of Newcastle Human Research Ethics Committee (H-2012-0246) and the Hunter New England Human Research Ethics Committee (HREC/12/HNE/140).

### 2.1. Study Design

The Protection Motivation Theory [15] proposes that health behaviors are the result of coping responses to perceived threats of vulnerability and severity. The theory posits that individuals look at the perceived benefits and usefulness of performing adaptive (helpful), and their confidence to perform them. Therefore, the intervention sought to capitalize on the awareness of personal risk, incorporate strategies to build self-efficacy for helpful tasks, and give meaningful feedback on performing the recommended strategies.

The study was a pilot parallel group randomized controlled trial. Participating families were stratified by sex of the index member, CVD event (stroke/ischemic heart condition) and time of event (≤6 months, >6 months) and randomized in blocks of six using QuickCalcs Software [16] to either the three-month intervention or feedback only control group, with assessors blinded to group allocation. Subjects received group assignment via the next available sealed envelope within their stratification.

**Figure 1:**
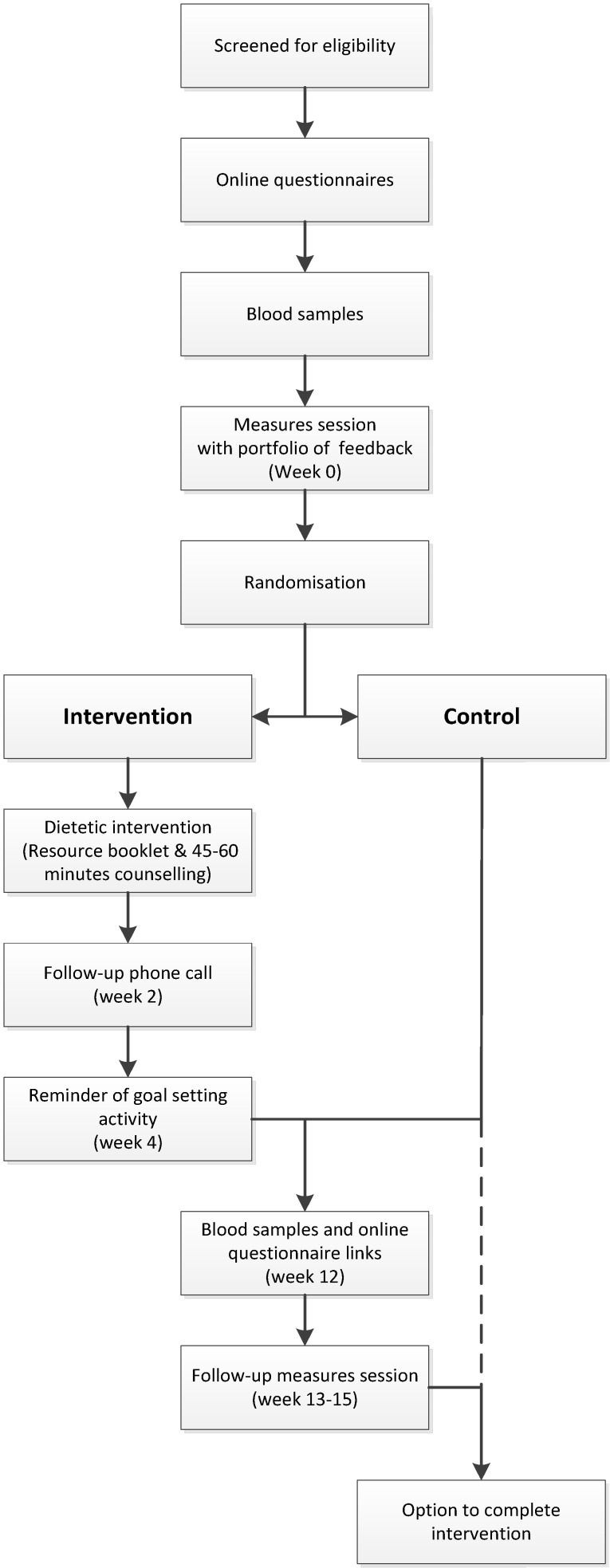
Intervention flow chart for study participants. Families randomized to the control group had the option of undergoing the intervention once the control period was completed.

Recruitment, baseline and follow-up assessments took place from December 2012 to May 2014. The intervention flow is summarized in Figure 1. After providing consent, families completed online questionnaires on demographics, medical history, smoking, and usual eating patterns. Fasting blood samples were analyzed for blood lipids prior to anthropometric assessments, and all individuals received a personalized feedback booklet containing lipid test results, anthropometric measures, and dietary intake analysis including macronutrient and micronutrient intakes and the percentage energy contributed by core (nutrient dense) and discretionary (energy-dense, nutrient-poor) foods. Randomization into intervention or control groups (feedback only) followed provision of the feedback booklet with those in the control group wait-listed for three months. Intervention group participants each received one 45-min dietary counselling session with an accredited practicing dietitian (APD). To ensure consistency of the intervention delivery, a resource booklet specific to the intervention and a semi-structured education session for the counselling were used, which allowed for modification of strategies to cater for families’ unique needs. Participants were asked to increase their intake of specific foods to more closely align with targets. The dietary intake targets used in the current intervention included: up to two serves (60 g) of nuts per day; 2–3 daily serves (2–3 g) of plant sterols; up to five daily serves (15 g) of soluble fibers; up to seven daily (42 g) serves of soy proteins; 2–3 serves of fish per week (170–450 g, dependent on fish type); up to seven serves (approximately 650 g) of legumes/pulses/lentils per week. Unsaturated fats were promoted whilst reducing saturated fats, as well as low-sodium food choices and general healthy eating guidelines [17].

### 2.2. Dietary Change Measures

Dietary intake was assessed as frequency of usual intake over the past six months using the validated Australian Eating Survey (≥18 years) (AES) and Australian Child and Adolescent Eating Survey (ACAES), 120 item semi-quantitative food frequency questionnaires (FFQs) [18,19]. Nutrient intakes were computed using FoodWorks version 4.00.1158 [20] and the Australian AusNut 1999 nutrient database (All Foods, Revision 17) and AusFoods (Brands, Revision 5). Foods specific to the Mediterranean [6] and Portfolio diets [7] not included within the AES and ACAES were measured using a 72 question semi-quantitative FFQ that was specifically developed to assess intake of plant sterols, viscous fibers, soy proteins and provide specific details about fat type, sodium, legumes, nuts and fish intakes. The study-specific FFQ was comprised of 18 food habit questions and 35 intake questions with stated serve sizes aligned to the Australian Dietary Guidelines [21], Heart Foundation recommendations [22], or as natural portion sizes and 19 questions with portion size stated as “1 serving”.

### 2.3. Qualitative Measures to Assess Feasibility, Dietary Changes and Program Acceptability

All adult family members completing the intervention were invited by post to participate in a semi-structured telephone interview. Areas of enquiry explored by interview included motivation to participate, barriers to healthy eating and dietary changes made. Individuals were interviewed by a female research team member (TS) 1–6 months (mean 1.5 months) post completion using a semi-structured discussion framework developed by the researchers. Probes and prompts were used to expand and clarify responses. The interviews were digitally recorded with the participants’ consent and transcribed verbatim. A computer program (NVIVO 10) was used to assist with the organizational aspects of data analysis. Qualitative analyses were conducted by an independent experienced qualitative researcher who was not part of the research team to reduce bias and ensure accuracy of themes identified. All index participants were asked to complete a process evaluation questionnaire after completing the three-month intervention. Questions were in regards to the suggested foods and eating patterns, resources provided, changes in behavior and general feedback.

### 2.4. Other CVD Related Health Measures

Participants’ height and weight were measured in light clothing to 0.1 cm and 0.1 kg, respectively using the Biospace BSM370 Automatic BMI Scale Stadiometer (Biospace Co. Ltd., Seoul, Korea). Waist circumference was measured at the narrowest point between the lower costal (10th rib) border and the top of the iliac crest using a non-extensible steel tape (KDFS10-02, KDS Corporation, Osaka, Japan). Brachial and central blood pressure and arterial stiffness measures (augmentation index) were obtained with the Pulsecor Cardioscope II (Pulsecor Ltd., Auckland, New Zealand) using WelchAllyn FlexiPort reusable blood pressure cuffs. Participants were seated for five minutes before the first measurement occurred, and repeat measures were taken at two-minute intervals. Participants under the age of 18 years were also provided with a familiarization trial measure to reduce potential anxiety associated with this measurement. Physical activity in adults was assessed using the International Physical Activity Questionnaire long form (IPAQ) for the previous seven days.

Blood samples were assayed for markers of insulin resistance, inflammation and blood lipid concentrations (see Table 1) from adult family members after an overnight fast by trained phlebotomists and analyzed at a single accredited (National Association of Testing Authorities) pathology service.

**Table 1:** Baseline characteristics of the Love your food, Love your heart, Love your family study participants, inclusive of 15 families, presented as mean ± standard deviation, except where indicated.

	Children (<18 years)	Adults		All adults
	100% Female (*n* = 3)	Males (*n* = 20)	Females (*n* = 21)	(*n* = 41, 100%)
Age (years) median (p25–p75)	12.9 (7.9–16.7)	59.4 (46.0–67.8)	56.6 (42.7–64.0)	59.0 (42.7–66.5)
Height (cm)	151.1 ± 22.0	174.8 ± 6.1	162.8 ± 6.2	168.6 ± 8.6
Weight (kg)	41.9 ± 15.1	87.7 ± 15.6	75.9 ± 18.9	81.6 ± 18.2
BMI (kg/m^2^)	17.8 ± 2.1	28.7 ± 5.1	28.6 ± 7.2	28.7 ± 6.2
Waist (cm)	60.7 ± 6.5	99.5 ± 15.1	86.9 ± 12.2	93.1 ± 14.9
Brachial BP (mmHg)				
systolic	100.3 ± 7.4 ^a^	121.3 ± 15.6	114.3 ± 15.0	117.7 ± 15.5
diastolic	63.5 ± 7.1 ^a^	71.8 ± 6.7	71.7 ± 7.8	71.7 ± 7.2
Central BP (mmHg)				
systolic	91.8 ± 7.4 ^a^	114.1 ± 16.2	109.0 ± 14.7	111.5 ± 15.5
diastolic	65.3 ± 6.0 ^a^	73.5 ± 7.0	72.8 ± 7.9	73.1 ± 7.4
Arterial stiffness	43 ± 11 ^a^	75 ± 34	86 ± 35	81 ± 35
Level of physical activity ^b^				
Low	N/A	10% (*n* = 2)	19% (*n* = 4)	15% (*n* = 6)
Moderate	N/A	60% (*n* = 12)	62% (*n* = 13)	61% (*n* = 25)
High	N/A	30% (*n* = 6)	19% (*n* = 4)	24% (*n* = 10)
Smoking status				
Current	N/A	*n* = 2 (10%)	*n* = 0 (0%)	*n* = 2 (5%)
Previous	N/A	*n* = 4 (20%)	*n* = 7 (33%)	*n* = 11 (27%)
Blood biomarkers				
Triglycerides (mmol/L)	N/A	1.3 ± 0.6	1.4 ± 0.9	1.3 ± 0.8
TC (mmol/L)	N/A	4.5 ± 1.1	5.3 ± 1.1	4.9 ± 1.2
LDL (mmol/L)	N/A	2.7 ± 1.1	3.3 ± 1.1	3.0 ± 1.1
HDL (mmol/L)	N/A	1.2 ± 0.3	1.5 ± 0.3	1.3 ± 0.4
Total: HDL ratio	N/A	3.9 ± 1.5	3.8 ± 1.0	3.9 ± 1.2
BGL (mmol/L)	N/A	5.0 ± 0.5	5.2 ± 0.8	5.1 ± 0.6
Insulin (IU/L)	N/A	8.8 ± 4.6	7.0 ± 3.4	7.9 ± 4.1
hsCRP (mg/L)	N/A	3.0 ± 3.4	2.4 ± 2.4	2.7 ± 2.9
ALT (U/L)	N/A	32.6 ± 10.8	22.5 ± 16.1	27.4 ± 14.5
AST (U/L)	N/A	30.0 ± 8.4	27.4 ± 25.8	28.7 ± 19.2
GGT (U/L)	N/A	28.0 ± 16.4	25.7 ± 25.6	26.8 ± 21.4

Abbreviations: BMI—Body mass index; Waist—waist circumference; BP—blood pressure; TC—total cholesterol; LDL—LDL cholesterol; HDL—HDL cholesterol; BGL—blood glucose level; hsCRP—high sensitivity C-Reactive Protein; ^a^ *n* = 2; ^b^ As categorized by the International Physical Activity Questionnaire.

Recruitment and retention data were measured as those enrolling and completing the intervention and by qualitative interview. Changes in dietary intakes, as measured by FFQ, are presented as mean ± standard deviation for normally distributed data and median (p25–p75) for non-normal data. An intention-to-treat analysis was used with last observation carried forward for missing data. As this was a feasibility trial, power calculations were not performed. Dietary intake themes from qualitative interviews were reported. Results for acceptability of the prevention program are summarized from program evaluation questionnaires.

## 3. Results

### 3.1. Study Participants

**Figure 2:**
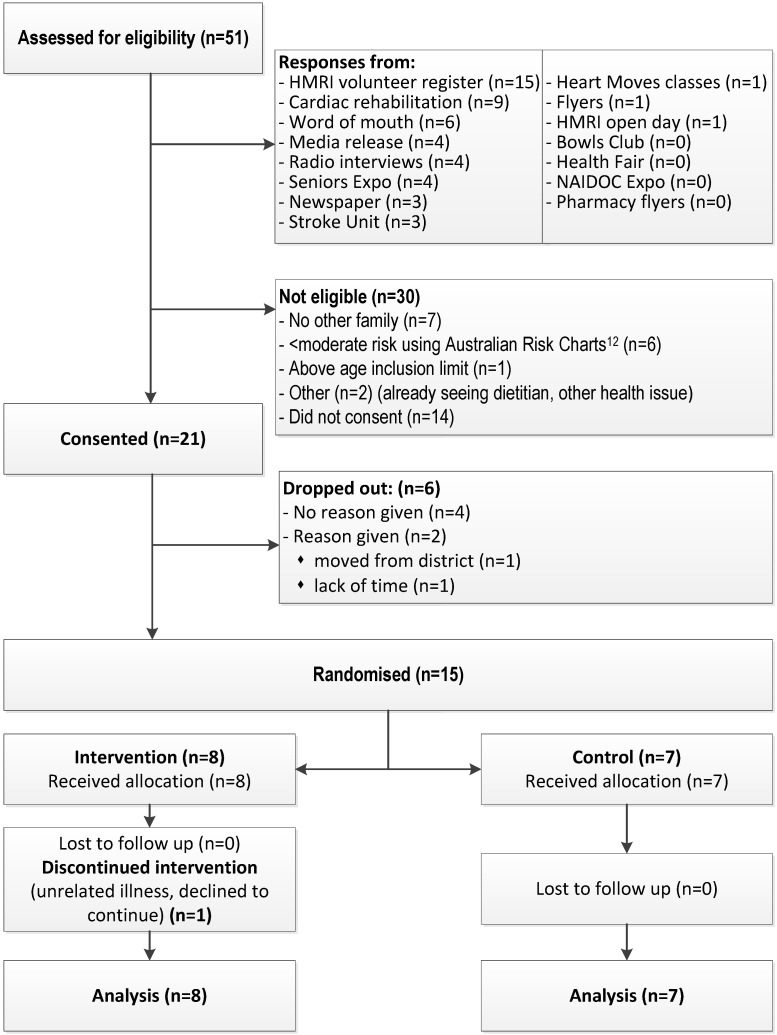
Flow chart showing the recruitment strategies used and number of participants assessed for eligibility and study retention.

Twenty-one index participants enrolled with their families, totaling 59 participants across three generations. Fifteen families were retained until randomization, consisting of 41 adults and three children (Figure 2). Of the 39 adults who completed the main study, 16 adults from eight families (41%) plus one child who turned 18 during the study participated in qualitative interviews (age range 18–70 years, 47% male). Five index participants were interviewed and one other had a diagnosed CVD condition. The interviews indicated participant motivations to join the study included a long-term interest in improving diet, a desire to make positive changes in eating habits and health for self and extended family, and having existing heart health issues. Individual participants identified a key family member who drove their family’s involvement, who was not necessarily the person with a CVD diagnosis.

Characteristics of the participants are summarized in Table 1. Sixteen participants (39%) reported knowing they had elevated serum cholesterol levels, with 18 (44%) taking lipid lowering medication. Twelve reported having high blood pressure (29%), with 17 (41%) on medication for this condition. Twelve (29%) had arthritis, with six taking medication (15%) and one had type 2 diabetes (medicated). Eleven of the 15 index recruits had experienced a prior CVD event; nine had been advised to attend cardiac rehabilitation, with seven having attending.

### 3.2. Feasibility of Recruiting and Retaining Families

Recruitment using a variety of methods (Figure 2) resulted in 51 index participants being assessed for eligibility over 16 months. Of 51 inquiries, 16 were not eligible and 14 did not return consent forms with the majority of those not returning consent forms recruited from cardiac rehabilitation and stroke units (*n* = 6) (Figure 2). Highest enrolment rates came from word-of-mouth (50%). Retention rates were highest (nine eligible, nine consented, seven completions) among those recruited from the Hunter Medical Research Institute volunteer register and media releases, and lowest among those recruited from cardiac rehabilitation classes or stroke units (11 eligible, five consents, two completions).

### 3.3. Nature and Extent of Dietary Changes Made

Baseline dietary intakes indicate that 63 ± 10% of energy came from nutritious, low energy-density (core) foods and 37 ± 10% from energy-dense, nutrient-poor (discretionary) foods. There was no difference in reported total energy intake at baseline between the adults or children completing the study and those who did not (*p* = 0.34 and *p* = 0.32 respectively). Analysis of dietary intakes and key components of the Mediterranean and Portfolio Diets are summarized in Table 2. Mean time between baseline and follow up was 4.5 months (±1.1). Table 3 summarizes foods habits relating to CVD health and highlights reductions in full-fat types of dairy and meat products usually eaten in the intervention group. Results at three-month follow up indicate that both groups made changes to their dietary intakes. The proportion of energy from core food groups showed improvement, as did daily vegetable intakes (Table 2).

**Table 2:** Baseline and follow-up dietary intakes as assessed by the Australian Eating Survey (AES), Australian Child and Adolescent Eating Survey (ACAES) and additional food frequency questionnaire assessing foods specific to the Mediterranean and Portfolio diets. Data presented as mean ± standard deviation for 15 families.

	Baseline				Follow up			
	Children	Adults			Children	Adults		
	(*n* = 3)	Adults (*n* = 41)	C (*n* = 18)	I (*n* = 23)	(*n* = 3)	Adults (*n* = 41)	C (*n* = 18)	I (*n* = 23)
Energy (kJ)	9967 ± 1619	10108 ± 2873	9582 ± 3197	10520 ± 2589	9565 ± 1942	9604 ± 2661	9253 ± 2878	9878 ± 2509
PE core foods (%)	57 ± 23	63 ± 10	66 ± 14	61 ± 6	63 ± 7	66 ± 11	68 ± 13	64 ± 9
PE discretionary foods (%)	43 ± 23	37 ± 10	34 ± 14	39 ± 6	37 ± 7	34 ± 11	32 ± 13	36 ± 9
PE protein (%)	20 ± 8	19 ± 4	21 ± 5	18 ± 2	20 ± 3	19 ± 3	21 ± 4	18 ± 2
PE CHO (%)	43 ± 8	44 ± 8	40 ± 8	47 ± 5	46 ± 8	43 ± 7	39 ± 7	47 ± 6
PE fats (%)	38 ± 1	33 ± 4	34 ± 4	32 ± 4	35 ± 7	33 ± 5	35 ± 6	31 ± 3
PE sat. fats (%)	18 ± 2	13 ± 2	14 ± 2	13 ± 2	15 ± 4	13 ± 2	14 ± 2	13 ± 2
Fiber (g)	25 ± 8	29 ± 11	28 ± 13	30 ± 8	27 ± 9	30 ± 9	27 ± 8	32 ± 9
Sodium (mg)	2067 ± 402	2321 ± 679	2197 ± 614	2418 ± 724	2067 ± 244	2259 ± 671	2275 ± 715	2246 ± 650
Fruit/day	2.0 ± 1.1	2.0. ± 1.7	1.8 ± 2.1	2.2 ± 1.3	2.4 ± 1.6	1.9 ± 1.1	1.5 ± 0.7	2.2 ± 1.2
Vegetables/day	4.6 ± 1.2	4.9 ± 2.1	5.2 ± 2.2	4.7 ± 2.1	4.3 ± 0.4	5.5 ± 1.7	5.6 ± 1.7	5.4 ± 1.7
ARFS ^a^	28 ± 8	35 ± 10	35 ± 8	35 ± 12	29 ± 6	39 ± 9	37 ± 5	39 ± 9
Frequency of foods specific to cardiovascular health (number of serves per day)								
Fat from added sources								
Saturated	N/A	0.3 ± 0.5	0.3 ± 0.7	0.2 ± 0.3	N/A	0.3 ± 0.7	0.5 ± 0.9	0.2 ± 0.3
Unsaturated	N/A	1.3 ± 0.8	1.4 ± 0.9	1.1 ± 0.8	N/A	1.2 ± 0.8	1.3 ± 0.9	1.1 ± 0.8
Nuts	0.2 ± 0.3	0.5 ± 0.6	0.5 ± 0.6	0.5 ± 0.5	1.1 ± 1.6	1.0 ± 1.1	0.9 ± 0.9	1.1 ± 1.2
Fish	0.3 ± 0.4	0.4 ± 0.5	0.5 ± 0.6	0.3 ± 0.3	0.5 ± 0.7	0.4 ± 0.3	0.5 ± 0.4	0.4 ± 0.3
Soy proteins	0.4 ± 0.7	0.1 ± 0.3	0.2 ± 0.4	0.1 ± 0.2	0.2 ± 0.3	0.2 ± 0.4	0.1 ± 0.3	0.3 ± 0.4
Legumes	0.1 ± 0.1	0.3 ± 0.4	0.3 ± 0.3	0.2 ± 0.5	1.2 ± 1.9	0.5 ± 0.7	0.7 ± 0.9	0.4 ± 0.5
Viscous fibers	0.2 ± 0.2	0.7 ± 0.7	0.7 ± 0.8	0.6 ± 0.6	0.3 ± 0.5	0.7 ± 0.7	0.4 ± 0.6	0.9 ± 0.8
Plant sterols	N/A	0.9 ± 1.1	1.2 ± 1.4	0.6 ± 0.9	N/A	0.7 ± 1.0	0.3 ± 0.7	1.0 ± 1.2

Abbreviations: C—Control group; I—Intervention group; N/A—Not assessed; PE—Percentage energy; ARFS—Australian Recommended Food Score; ^a^ measure of dietary variety.

**Table 3:** Reported eating habits of foods related to cardiovascular disease (CVD) health.

	Baseline			Follow up		
	All (*n* = 44)	C (*n* = 20)	I (*n* = 24)	All (*n* = 44)	C (*n* = 20)	I (*n* = 24)
Type of milk normally consumed						
Don’t drink milk	7%	0%	13%	7%	0%	13%
Normal	25%	15%	33%	14%	15%	13%
Reduced fat	32%	30%	33%	43%	40%	46%
Skim	32%	50%	17%	34%	40%	29%
Other	5%	5%	4%	2%	5%	0%
Type of cheese normally eaten						
Don’t eat this	7%	5%	8%	7%	5%	8%
Normal	55%	40%	67%	45%	45%	46%
Reduced fat	34%	55%	17%	32%	30%	33%
Low fat	2%	0%	4%	14%	15%	12%
Not sure	2%	0%	4%	2%	5%	0%
Type of meat						
Don’t eat this	2%	5%	0%	5%	10%	0%
Normal	55%	65%	46%	45%	65%	29%
Reduced fat	30%	10%	46%	39%	15%	58%
Low fat	11%	15%	8%	11%	10%	13%
Not sure	2%	5%	0%	0%	0%	0%
Type of chicken						
Don’t eat this	7%	5%	8%	7%	5%	8%
Fried	2%	0%	4%	0%	0%	0%
Crumbed	9%	5%	13%	7%	10%	4%
With skin	32%	40%	25%	25%	25%	25%
Skin removed	45%	45%	46%	55%	55%	54%
Not sure	5%	5%	4%	7%	5%	8%
Adding of salt to food						
Never add salt	25%	25%	25%	16%	15%	17%
During cooking	32%	35%	29%	36%	45%	29%
To meals	27%	30%	25%	25%	15%	33%
Both meals & cooking	14%	5%	21%	20%	20%	21%
Not sure	2%	5%	0%	2%	5%	0%
Take away per week *						
None	9%	15%	4%	11%	15%	8%
<once per week	48%	45%	50%	57%	50%	62%
1–2 per week	36%	25%	46%	30%	30%	29%
3–4 per week	7%	15%	0%	2%	5%	0%
>4 per week	0%	0	0%	0%	0%	0%

Abbreviations: C—Control group; I—Intervention group; * Take away described as chinese, fish and chips, hamburgers and chips/fries, pizza.

Results from the qualitative interviews indicate that prior to program involvement 14 of 17 participants rated the healthiness of their diet subjectively as 6–7 on an alpha-numeric rating scale, where 10 represents the most healthy. Only two participants rated their pre-study diets as below average at three out of 10. Participants appeared to use a cognitive balancing of ‘good’ *versus* ‘bad’ aspects of their diet to justify their ratings of their usual intake pre-study. Dietary habits they acknowledged as reducing their ‘healthiness rating’ included the consumption of fatty meats, low vegetable intakes, and snacking on sugary foods between meals. Dietary habits perceived as increasing their ‘healthiness rating’ were cutting back on red meat by eating chicken and fish, and exercising ‘dietary moderation’ described as ‘nothing in excess’. These habits were perceived by some as making their diet healthier relative to a subjective ‘average’ to which they mentally compared their intakes. Although almost a third of participants acknowledged little change to the healthiness of their diet post-study due to persistence of major barriers (e.g., partner reluctance, personal preference and taste), the majority reported having made permanent changes to their dietary intake and food habits. Some households reported a subjective rating improvement of 2–3 out of 10 post-program participation, suggesting substantial changes were made.

These improvements were attributed to increased knowledge and awareness due to program participation and appeared to inspire greater experimentation with healthier options and purchasing of foods reflecting increased variety and nutritional quality.

“I decided I would make a lovely rice dish, and I put in some slivered almonds and a couple of herbs and some garlic and it was lovely, and a little bit of soy sauce…I think the main thing is, after this study, was just variety. Like, if I was to make a rice dish before that I wouldn’t have thought to add in nuts.”

Further examples of dietary improvements given were less impulsive food shopping, more variety in fruit and vegetable selection, lower sugar and fat options, use of legumes, lentils and soy products, healthier meat options, and elimination of energy-dense, nutrient-poor foods. For all participants, including those reporting little or no change in their diet post-study, involvement in the project appeared to have increased awareness of the different components of their diet. Examples given included the proportion of energy from discretionary foods, foods with a healthy heart tick, the healthiness of different types of fat, an increased awareness of processed foods and the importance of small changes. Indeed, one participant who only reported slight changes in his diet following the study described the cumulative impact of these small changes as evidence of a shift in his food behaviors and preferences:

“I just cut out more of the bad stuff, like I’m sort of thinking it was only marginal changes I made. Look when I ate poorly like snacks and things like that, I'd probably eat too much. Whereas when I have a snack or a treat now, actually I find that I can’t eat as much anyway of it. I think my taste buds have changed a little bit. But again from the converse side of things, previously when I probably didn’t eat as much good food. I’m eating more good food now…It’s just those marginal shifts.”

### 3.4. Acceptability of Program to Align Current Eating Patterns with Recommendations

Eleven of the 15 index participants (73%) returned program evaluation forms. These participants all agreed or strongly agreed that this type of diet was relevant to them, but they had mixed responses regarding the ease of integration into their lives (55% positive, 18% negative, 27% neutral). Six (55%) felt it impacted negatively on grocery costs. Ten participants (91%) agreed or strongly agreed they would recommend this type of eating pattern to people in a similar situation (*n* = 1 neutral). Ten (91%) found the resource booklet easy to read and the information easy to understand, with the remainder (*n* = 1) answering neutral to both questions. Nine of the eleven participants (82%) read the booklet 2–3 or ≥3 times, with two participants (18%) reading it once. The individualized feedback booklets were similarly valued with eight participants (73%) reading it 2–3 or ≥3 times and three participants (27%) reading it once.

## 4. Discussion

The current study investigated whether families could be recruited and retained in a family CVD prevention program that was based on the Mediterranean and Portfolio eating patterns. Recruitment was challenging, with only 15 of 35 eligible families who initially expressed interest, engaging with the study through to the randomization stage. However, once randomized, the majority of these families completed the intervention. Those responding to media releases about the study and volunteer register invitations were more likely to be retained. Of interest is that amongst those families completing the trial, a key family member was found to drive the involvement and retention of the family. While overall dietary patterns were unaltered, participants made small, but incremental dietary changes, such as reducing discretionary foods and selecting fat-reduced versions of milk and cheese and fat-trimmed meats. Participants reported an increased awareness of their food habits and knowledge of food following the personalized dietary counselling they received from the study dietitian about their usual food and nutrient intakes. Evaluation of the program found that although participants noted some negatives, such as increases in grocery costs, these may have been offset by reductions in costs associated with takeaway foods. Evaluation of food costs in future studies is required. Participants used the resources and dietary feedback provided on multiple occasions and reported they would recommend the program to others in a similar situation.

A clear barrier to recruitment occurred between confirming eligibility of the index participant and the returning of consent forms from the family group, as shown by the limited number of returned consents (*n* = 14) at this stage. This suggests that persuading a family member to participate was a substantial barrier. An additional seven interested participants were deemed ineligible because they could not identify a family member to accompany them in the study. A larger Canadian family-based study had a similar focus, but recruited at-risk family members (*n* = 426) through in-patients from a tertiary care cardiac center [23]. While this study was able to randomize a greater proportion of their eligible participants, it had a 26% loss to follow-up. Recruiting using these methods may capitalize on a teachable moment, and lead to a change in lifestyle intentions [24], but does not necessarily imply a willingness to make permanent lifestyle changes amongst family members.

The lack of perceived CVD risk amongst those with actual increased risk is a significant barrier to program uptake as identified in the current study. The Protection Motivation Theory, on which the current study is based, identifies that a perception of risk must be present before any change in behavior can occur [15]. However, risk of CVD events is often poorly perceived by those with a confirmed family history of CVD, and may not be sufficient to change or act on intentions suggesting other motivators are required [25]. In the current study, some individuals lacked understanding of their medical risk factors, evidenced by the large proportion who were currently taking medications for lipid lowering or blood pressure control, but who did not identify when asked whether they had these conditions or any medical problems. Addressing appropriate awareness and management of risk is likely to be an important component in engaging people in CVD prevention programs. Future studies should consider identifying and engaging a key family member capable of influencing other family members. The recruitment approach for this study used the index person as the primary contact for the family in the first instance, but it may have been more advantageous to allow a key family member to engage on behalf of the high-risk participant.

The dietary components of this study were modelled on the Mediterranean and portfolio eating patterns as these have been shown to be efficacious in reducing CVD risk. Participants commencing the study reported dietary patterns that did not align well to these eating patterns and had higher than recommended intakes of discretionary food choices. Comparison of the dietary intakes of participants in the current study to data from the 2011–2012 Australian Health Survey (AHS) [26] indicates that this group were consuming higher energy intakes compared to the national average of 8672 kJ (value also obtained from 24-h recall), both before and after the intervention, while the proportion of energy from discretionary food choices was similar at 34.6% of total energy for adults. The macro-nutrient contributions appeared unchanged by the intervention and appears comparable to the national average, although small differences can be seen between the control and intervention groups. There was no apparent change in saturated fat intakes as analyzed by the FFQ, although questions on dietary habits (Table 3) indicate that saturated fat may have been decreased through the choosing of different cuts of meats. Within both this study and in a study of 426 family members of coronary artery disease patients by Reid *et al.* [23], participants were only able to make small increases in intakes of vegetables, showing this to be an area to be addressed in future work. Individually tailored dietary counselling immediately after personal dietary and risk biomarker feedback in the current study resulted in favorable changes in terms of selecting lower fat dairy products and fat-trimmed meat products, which may be due to capitalizing on the teachable moment the personalized feedback helped to facilitate. A possible strategy to enhance adherence in future studies includes the provision of feedback in an educational context, based on measured anthropometrics and blood lipids at an interim stage following initial dietary modifications, instead of at the end of the study as given here which may have increased motivation. Participants were contacted by a single telephone call during the three months follow-up period to discuss any difficulties they had encountered and to encourage maintenance of dietary changes made. This level of engagement was chosen and was comparable to a longer study by Jenkins *et al.* [27], which showed that more intensive follow-up did not greatly improve adherence in this type of diet.

The limitations of the current study include the recruitment of a small non-representative sample of families who volunteered. There may have been a seasonality bias influencing the reported dietary intakes impacting on both the control and intervention groups. The dietary modifications made may not be sufficient to show clinically important and statistically significant changes in serum lipids in the short term, but may benefit the individuals if continued long term [28] and a larger study with longer follow-up would be needed to evaluate this.

## 5. Conclusions

While the goal of primary prevention is to avert disease in high-risk individuals, the current study highlights there is little motivation to participate in CVD prevention programs when risk is poorly perceived and therefore insufficient to prompt behavior change. The program structure in the current study demonstrated promising results, but the challenges of recruitment need to be overcome. Once engaged, families were willing and able to make small incremental change in their dietary choices associated with CVD risk reduction in the long-term. Further research is needed to identify CVD-related motivators of dietary change, particularly those that engage individuals and have the ability to engage all family members in improving health behaviors.

## References

[1] World Health Organization (2015). Global Status Report on Noncommunicable Dieases 2014.

[2] World Health Organization (2011). Global Atlas on Cardiovascular Disease Prevention and Control.

[3] Kral T.V.E., Rauh E.M. (2010). Eating behaviors of children in the context of their family environment. Physiol. Behav..

[4] Lloyd-Jones D.M., Nam B.H., D’Agostino R.B., Levy D., Murabito J.M., Wang T.J., Wilson P.W., O’Donnell C.J. (2004). Parental cardiovascular disease as a risk factor for cardiovascular disease in middle-aged adults: A prospective study of parents and offspring. JAMA.

[5] Bao W., Srinivasan S.R., Valdez R., Greenlund K.J., Wattigney W.A., Berenson G.S. (1997). Longitudinal changes in cardiovascular risk from childhood to young adulthood in offspring of parents with coronary artery disease: The Bogalusa heart study. JAMA.

[6] Estruch R., Ros E., Salas-Salvadó J., Covas M.I., Corella D., Arós F., Gómez-Gracia E., Ruiz-Gutiérrez V., Fiol M., Lapetra J. (2013). Primary prevention of cardiovascular disease with a mediterranean diet. N. Engl. J. Med..

[7] Jenkins D.J.A., Josse A.R., Wong J.M.W., Nguyen T.H., Kendall C.W.C. (2007). The portfolio diet for cardiovascular risk reduction. Curr. Atheroscler. Rep..

[8] Pachucki M.A., Jacques P.F., Christakis N.A. (2011). Social network concordance in food choice among spouses, friends, and siblings. Am. J. Public Health.

[9] Jenkins D.J.A., Chiavaroli L., Wong J.M.W., Kendall C., Lewis G.F., Vidgen E., Connelly P.W., Leiter L.A., Josse R.G., Lamarche B. (2010). Adding monounsaturated fatty acids to a dietary portfolio of cholesterol-lowering foods in hypercholesterolemia. Can. Med. Assoc. J..

[10] Jenkins D.J.A., Kendall C.W.C., Faulkner D.A., Nguyen T., Kemp T., Marchie A., Wong J.M.W., de Souza R., Emam A., Vidgen E. (2006). Assessment of the longer-term effects of a dietary portfolio of cholesterol-lowering foods in hypercholesterolemia. Am. J. Clin. Nutr..

[11] Dalen J.E., Devries S. (2014). Diets to prevent coronary heart disease 1957–2013: What have we learned?. Am. J. Med..

[12] Huang J., Frohlich J., Ignaszewski A.P. (2011). The impact of dietary changes and dietary supplements on lipid profile. Can. J. Cardiol..

[13] Anderson T.J., Grégoire J., Hegele R.A., Couture P., Mancini G.B.J., McPherson R., Francis G.A., Poirier P., Lau D.C., Grover S. (2013). 2012 update of the Canadian cardiovascular society guidelines for the diagnosis and treatment of dyslipidemia for the prevention of cardiovascular disease in the adult. Can. J. Cardiol..

[14] National Heart Foundation of Australia (National Blood Pressure and Vascular Disease Advisory Committee) (2010). Guide to Management of Hypertension 2008. Updated December 2010.

[15] Armitage C.J., Conner M. (2000). Social cognition models and health behaviour: A structured review. Psychol. Health.

[16] GraphPad Software Inc. Quickcalcs. http://graphpad.com/quickcalcs/randomN1.cfm.

[17] NHMRC, Australian Government Australian Guide to Healthy Eating. http://www.eatforhealth.gov.au/guidelines/australian-guide-healthy-eating.

[18] Collins C.E., Boggess M.M., Watson J.F., Guest M., Duncanson K., Pezdirc K., Rollo M., Hutchesson M.J., Burrows T.L. (2014). Reproducibility and comparative validity of a food frequency questionnaire for australian adults. Clin. Nutr..

[19] Watson J.F., Collins C.E., Sibbritt D.W., Dibley M.J., Garg M.L. (2009). Reproducibility and comparative validity of a food frequency questionnaire for australian children and adolescents. Int. J. Behav. Nutr. Phys. Act..

[20] (2005). Xyris Software, version 4.00.1158.

[21] Department of Health and Aging, National Health and Medical Research Council, Australian Government (2013). Australian Dietary Guidelines.

[22] National Heart Foundation of Australia (2008). Fish, Fish Oils, n-3 Polyunsaturated Fatty Acids and Cardiovascular Health.

[23] Reid R.D., McDonnell L.A., Riley D.L., Mark A.E., Mosca L., Beaton L., Papadakis S., Blanchard C.M., Mochari-Greenberger H., O’Farrell P. (2014). Effect of an intervention to improve the cardiovascular health of family members of patients with coronary artery disease: A randomized trial. Can. Med. Assoc. J..

[24] Cohen D.J., Clark E.C., Lawson P.J., Casucci B.A., Flocke S.A. (2011). Identifying teachable moments for health behavior counseling in primary care. Patient Educ. Couns..

[25] Imes C.C., Lewis F.M. (2014). Family history of cardiovascular disease, perceived cardiovascular disease risk, and health-related behavior: A review of the literature. J. Cardiovasc. Nurs..

[26] Australian Bureau of Statistics (2014). 4364.0.55.007-Australian Health Survey: Nutrition First Results—Food and Nutrients, 2011–2012.

[27] Jenkins D.A., Jones P.H., Lamarche B., Kendall C.W.C., Faulkner D., Cermakova L., Gigleux I., Ramprasath V., de Souza R., Ireland C. (2011). Effect of a dietary portfolio of cholesterol-lowering foods given at 2 levels of intensity of dietary advice on serum lipids in hyperlipidemia: A randomized controlled trial. JAMA.

[28] Mozaffarian D., Hao T., Rimm E.B., Willett W.C., Hu F.B. (2011). Changes in diet and lifestyle and long-term weight gain in women and men. N. Engl. J. Med..

